# Assessing and Explaining Geographic Variations in Mammography Screening Participation and Breast Cancer Incidence

**DOI:** 10.3389/fonc.2019.00909

**Published:** 2019-09-18

**Authors:** Jonas Czwikla, Iris Urbschat, Joachim Kieschke, Frank Schüssler, Ingo Langner, Falk Hoffmann

**Affiliations:** ^1^Department of Health Services Research, Carl von Ossietzky University of Oldenburg, Oldenburg, Germany; ^2^Department of Health, Long-Term Care and Pensions, SOCIUM Research Center on Inequality and Social Policy, University of Bremen, Bremen, Germany; ^3^High-Profile Area of Health Sciences, University of Bremen, Bremen, Germany; ^4^Epidemiological Cancer Registry of Lower Saxony, Registry Unit Oldenburg, Oldenburg, Germany; ^5^Institute for Applied Photogrammetry and Geoinformatics, Jade University of Applied Sciences Wilhelmshaven/Oldenburg/Elsfleth, Oldenburg, Germany; ^6^Department of Clinical Epidemiology, Leibniz Institute for Prevention Research and Epidemiology – BIPS, Bremen, Germany

**Keywords:** breast cancer, mammography screening, participation, incidence, geographic variations, cancer registry data, screening unit data, health insurance claims data

## Abstract

Investigating geographic variations in mammography screening participation and breast cancer incidence help improve prevention strategies to reduce the burden of breast cancer. This study examined the suitability of health insurance claims data for assessing and explaining geographic variations in mammography screening participation and breast cancer incidence at the district level. Based on screening unit data (1,181,212 mammography screening events), cancer registry data (13,241 incident breast cancer cases) and claims data (147,325 mammography screening events; 1,778 incident breast cancer cases), screening unit and claims-based standardized participation ratios (SPR) of mammography screening as well as cancer registry and claims-based standardized incidence ratios (SIR) of breast cancer between 2011 and 2014 were estimated for the 46 districts of the German federal state of Lower Saxony. Bland-Altman analyses were performed to benchmark claims-based SPR and SIR against screening unit and cancer registry data. Determinants of district-level variations were investigated at the individual and contextual level using claims-based multilevel logistic regression analysis. In claims and benchmark data, SPR showed considerable variations and SIR hardly any. Claims-based estimates were between 0.13 below and 0.14 above (SPR), and between 0.36 below and 0.36 above (SIR) the benchmark. Given the limited suitability of health insurance claims data for assessing geographic variations in breast cancer incidence, only mammography screening participation was investigated in the multilevel analysis. At the individual level, 10 of 31 Elixhauser comorbidities were negatively and 11 positively associated with mammography screening participation. Age and comorbidities did not contribute to the explanation of geographic variations. At the contextual level, unemployment rate was negatively and the proportion of employees with an academic degree positively associated with mammography screening participation. Unemployment, income, education, foreign population and type of district explained 58.5% of geographic variations. Future studies should combine health insurance claims data with individual data on socioeconomic characteristics, lifestyle factors, psychological factors, quality of life and health literacy as well as contextual data on socioeconomic characteristics and accessibility of mammography screening. This would allow a comprehensive investigation of geographic variations in mammography screening participation and help to further improve prevention strategies for reducing the burden of breast cancer.

## Introduction

Organized population-based mammography screening programs aim to reduce the mortality of breast cancer (1, 2), which is accountable for most cancer related deaths among women (3). Screening-detected breast cancer at an early stage is assumed to result in more effective treatment options, a higher curative potential, and a better survival probability compared to breast cancer clinically detected in symptomatic women (4, 5).

After randomized trials had found that mammography screening can reduce breast cancer mortality (6–9), mammography screening was established in different variations in, among other countries, the United States of America (10, 11), Canada (12), Australia (13), and European Union member states (14–17). In Germany, the implementation of an organized population-based mammography screening program started in 2005 and was completed nationwide in 2009 (18–20). Currently, more than 95% of women aged 50–69 years are invited biennially to participate (21). In 2016, however, the German participation rate of 51% (21) was lower than the acceptable level of 70% defined in the European Guidelines (14) and also lower than the European average of 60% (22).

In Germany, as in other high-income countries (22, 23), spatial disparities persist in mammography screening participation. At the highest level of territorial division (i.e., 16 federal states of Germany), participation rates range from 44% in Bavaria and Berlin to 61% in Mecklenburg-Western Pomerania (21). Geographic variations at lower levels of territorial division in Germany (i.e., 294 rural and 107 urban districts, 11,054 municipalities), however, have hitherto rarely been investigated. Only at the neighborhood-level, one small-area spatio-temporal analysis of mammography screening participation rates in the City of Dortmund in western Germany found lower participation rates in socioeconomically deprived neighborhoods (24).

Investigating geographic variations in mammography screening participation can help to identify individual and contextual determinants of mammography screening and thus may help to improve this early detection strategy (25–27). To reduce the burden of breast cancer, however, not only effective early detection strategies but also effective primary prevention strategies aiming to reduce breast cancer incidence are required (3, 28). To be most effective, these strategies need to consider both individual and contextual determinants of breast cancer incidence (2, 3, 29). These determinants can also be investigated in the light of geographic variations (30–33).

Whereas screening unit and cancer registry data are commonly used for investigating geographic variations in mammography screening participation and breast cancer incidence (24, 30–35), the potential of health insurance claims data for such purposes has so far rarely been investigated (26, 27, 36). The particular advantage of health insurance claims data is that they comprise individual information on sociodemographic characteristics, in- and outpatient diagnoses and health care provision for both mammography screening participants and non-participants as well as for women with and without cancer. Furthermore, they offer the opportunity to link contextual data (e.g., on area deprivation) to insured persons, which might serve to gain a deeper picture on individual and contextual determinants of spatial disparities in mammography screening participation and breast cancer incidence.

Besides these advantages, a potential disadvantage of health insurance claims data is that they usually include a selective population (e.g., insured persons from only one health insurance fund) (37, 38). Furthermore, they comprise no information as to whether a cancer diagnosis is incident, prevalent or recurrent (39). In Germany, moreover, validation studies of health insurance claims data are urgently needed. This holds especially true since German health insurance claims data represent an important data source for the ongoing evaluation of breast cancer mortality in the German mammography screening program (19).

This study aimed to examine the suitability of health insurance claims data for assessing and explaining geographic variations in mammography screening participation and breast cancer incidence at the district level. The specific aims were (i) to assess district-level variations in mammography screening participation and breast cancer incidence in the German federal state of Lower Saxony using screening unit and cancer registry data, (ii) to assess the same district-level variations using health insurance claims data and (iii) to examine the potential of health insurance claims data linked to contextual district-level data for explaining the assessed district-level variations.

## Materials and Methods

### Data Sources

The study was based on pseudonymous health insurance claims data, anonymous screening unit and cancer registry data as well as district-level contextual data. Claims data were provided by the BARMER, one of the two largest nationwide German statutory health insurance funds covering 9.2 million individuals (i.e., 13% of 72.8 million statutorily health insured people in Germany). In Lower Saxony, the BARMER covers 900,000 individuals (i.e., 13% of 7.0 million statutorily health insured people in Lower Saxony). We considered claims data covering the years 2008 to 2015, comprising information on year of birth; sex; district of residence; start and end dates of insurance periods; in- and outpatient diagnoses coded according to the International Classification of Diseases, 10th Revision (ICD-10), German Modification; as well as outpatient procedures coded according to the German doctor's fee scale.

Screening unit data for Lower Saxony were provided by the eight screening units located in the federal state of Lower Saxony and comprised the number of mammography screening events in 2011, 2012, 2013, and 2014 stratified by district and age.

Cancer registry data for Lower Saxony were provided by the Epidemiological Cancer Registry of Lower Saxony and comprised the number of incident breast cancer (ICD-10 C50) cases (including death certificate only cases) registered in 2011, 2012, 2013, and 2014 (as of January 01, 2019) stratified by district and age. Contextual data for Lower Saxony comprising information on unemployment, income, education, foreign population and type of district (Supplementary Material) were publicly available from the Federal Institute for Research on Building, Urban Affairs and Spatial Development (40).

### Study Design and Populations

The cohort study was conducted in Lower Saxony. Located in northwest Germany, Lower Saxony has the second largest area and fourth largest population among all 16 German federal states. Currently, about 1,151,000 women residing in Lower Saxony are 50–69 years old and represent the target population of the German Mammography Screening Program in Lower Saxony. In the period analyzed, Lower Saxony consisted of 46 districts (including 8 urban districts and Hanover Region) each of which accounted for between 0.6 and 14.4% of the total population of Lower Saxony.

In our screening unit and cancer registry data-based analyses, all mammography screening events as well as all incident breast cancer cases (including death certificate only cases) in Lower Saxony in women aged 50–69 years between 2011 and 2014 were considered. In our claims-based analyses, women aged 50–69 years in 2011, 2012, 2013, or 2014, residing in one of the 46 districts of Lower Saxony and insured on at least 1 day between January 1, 2011 and December 31, 2014 were eligible for inclusion. Insured persons with invalid information on sex, age or district of residence (1.0% of the claims-based population) were not considered. The resulting population was further restricted according to the inclusion criteria of the respective analyses defined in section Statistical Analyses.

### Identification of Mammography Screening Participation and Breast Cancer Incidence in Health Insurance Claims Data

In health insurance claims data, mammography screening participants were identified on the basis of the specific outpatient procedure code 01750 for screening mammography. In a given period, women whose records contained this code were defined as participants and women without this code in their records were defined as non-participants.

Since health insurance claims data comprise no information as to whether a cancer diagnosis is incident, prevalent or recurrent (39), incident breast cancer cases were defined using the following algorithm:

- A diagnosis of malignant neoplasms of breast (ICD-10 C50) in a given year,
- no diagnosis of malignant neoplasms of breast and no diagnosis of a personal history of malignant neoplasms of breast (ICD-10 C50 and Z85.3) in 3 continuously insured years preceding the index-quarter, and
- a confirming second diagnosis of malignant neoplasms of breast (ICD-10 C50) in the subsequent quarter, or death in the index-quarter or following quarter.

### Statistical Analyses

#### Assessment of Geographic Variations

Standardized participation ratios (SPR) of mammography screening in the 46 districts of Lower Saxony were calculated for the total 4-years period covering the years 2011 to 2014. In the screening unit data-based analyses, for each year, the denominator comprised the total population of women aged 50–69 years (i.e., women not invited for participation as well as prevalent breast cancer cases remained in the denominator). The numerator comprised the number of mammography screening events among women aged 50–69 years in the respective year.

In the claims-based analyses, for each year, the denominator comprised the total population of insured women aged 50–69 years who were insured on July 01 of the respective year (i.e., mid-year population). The numerator comprised the number of insured women aged 50–69 years identified as mammography screening participants in the respective year.

In both the screening unit data and claims-based analyses, SPR were calculated in three steps: First, age-specific (5-years age groups) participation rates in overall Lower Saxony in the total 4-years period were calculated. The rates were calculated on the basis of age-specific numbers of mammography screening events in the total 4-years period (numerator) and the sums of the age-specific populations of women in 2011, 2012, 2013, and 2014 divided by two to take account of the biennial invitation rounds (denominator). Second, for each district, the expected age-specific numbers of mammography screening events in the total 4-years period were calculated on the basis of age-specific participation rates in Lower Saxony and the sums of the age-specific populations of women in the respective district in 2011, 2012, 2013, and 2014 divided by two to take account of the biennial invitation rounds. Finally, SPR were calculated by dividing the observed by the expected total number of mammography screening events in a district. 95% confidence intervals (CI) for SPR were calculated on the basis of Byar's approximation (41). For example, a SPR of 1.2 indicates that the observed number of mammography screening events in a district is 20% higher than expected.

Using a Bland-Altman analysis (42, 43), claims-based SPR were benchmarked against screening unit data-based SPR. In the Bland-Altman analysis, the differences between claims and screening unit data were plotted against the average of claims and screening unit data in SPR. 95% limits of agreement (LoA) were calculated according to the method proposed by Bland and Altman (43).

Standardized incidence ratios (SIR) of breast cancer in the 46 districts of Lower Saxony were also calculated for the total 4-years period covering the years 2011–2014. In the cancer registry data-based analyses, for each year, the denominator comprised the total population of women aged 50–69 years (i.e., prevalent breast cancer cases remained in the denominator which is in line with the methods used by the German Centre for Cancer Registry Data). The numerator comprised the number of incident breast cancer cases aged 50–69 years in the respective year.

In the claims-based analyses, for each year, the denominator comprised the total population of insured women aged 50–69 years who were continuously insured for the 3 years preceeding July 01 of the respective year (i.e., mid-year population). The numerator comprised the number of insured women aged 50–69 years identified as incident breast cancer cases in the respective year.

In the cancer registry data and claims-based analyses, SIR were calculated similar to SPR. In step 1, age-specific incidence rates in overall Lower Saxony were calculated on the basis of age-specific numbers of incident breast cancer cases and the sums of the age-specific populations of women in the 4-years period. In step 2, for each district, the expected age-specific numbers of incident breast cancer cases were calculated on the basis of age-specific incidence rates in Lower Saxony and the sums of the age-specific populations of women in the respective district in the 4-years period. Finally, SIR were calculated and claims-based SIR were benchmarked against cancer registry data-based SIR using the same methods as used for SPR.

Maps of district-level variations in SPR and SIR in Lower Saxony were created using QGIS 3.4.

#### Explanation of Geographic Variations

A claims-based multilevel logistic regression analysis with random intercepts only in which insured women were nested within districts was conducted. Since the suitability of health insurance claims data for assessing geographic variations in breast cancer incidence appeared to be limited, only the outcome mammography screening participation was analyzed. We defined a cohort including all women aged 50–66 years in 2011, who were continuously insured in the 3 years preceding January 01, 2011 and had no breast cancer diagnosis (ICD-10 C50 and Z85.3) and no diagnosis of carcinoma *in situ* of breast (ICD-10 D05) in 2008, 2009, and 2010 (i.e., women older than 69 in 2014 as well as women with breast cancer or carcinoma *in situ* of breast diagnoses, and thus not eligible for mammography screening participation, were not considered). Mammography screening participants and non-participants were identified between 2011 and 2014.

At the individual level, explanatory variables were age group in 2011 (50–54, 55–59, 60–64, 65–66 years) and the individual Elixhauser comorbidities (44) as described in the Supplementary Material. Furthermore, the end of follow-up (end of insurance period in 2011, 2012, 2013, 2014, respectively, end of study period on December 31, 2014) was considered as control variable for different observation times.

At the district level, explanatory variables were unemployment rate, average household income per inhabitant, proportion of employees without a qualification, proportion of employees with an academic degree, proportion of foreign population and type of district. With the exception of type of district, all contextual variables were categorized into quintiles (Supplementary Material).

Univariable logistic regression analyses were conducted to identify individual and contextual variables associated with mammography screening participation. Only explanatory variables with a *p*-value <0.2 (for variables with more than two categories with a *p*-value <0.2 in at least one category) were further considered. Multicollinearity between explanatory variables was assessed using the variance inflation factor with a cutoff value of 10 (45).

Multilevel regression with the district clustering variable was conducted (i) without individual- and contextual-level variables (empty model), (ii) including only individual-level variables as fixed effects and (iii) including both individual- and contextual-level variables as fixed effects. In all models, district-level variances and median odds ratios (MOR) were considered as measures of variation (46, 47). Proportional change in variance was calculated to evaluate changes in district-level variance between models 2 and 1, and 3 and 2 (46). The MOR was defined as the median odds ratio one insured women would have when moving between two randomly chosen districts to the district with a higher probability of participating in mammography screening (46). For example, a MOR of 1.0 indicates that there is no district-level variance in the probability of participating in mammography screening, and a MOR larger than 1.0 indicates that there is district-level variation (46, 47). Model fit was assessed using −2 log likelihood and compared between models 2 and 1, and 3 and 2 using the likelihood ratio test (48). The multilevel regression analyses were conducted using SAS Enterprise Guide 7.1. PROC GLIMMIX (48).

## Results

### Standardized Participation Ratios

**Figure 1:**
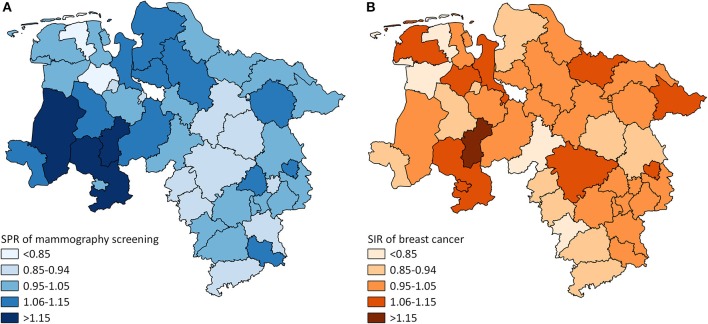
**(A)** District-level variation in standardized participation ratios (SPR) of mammography screening in Lower Saxony between 2011 and 2014 for women aged 50–69 years (screening unit data). **(B)** District-level variation in standardized incidence ratios (SIR) of breast cancer in Lower Saxony between 2011 and 2014 for women aged 50–69 years (cancer registry data).

**Figure 2:**
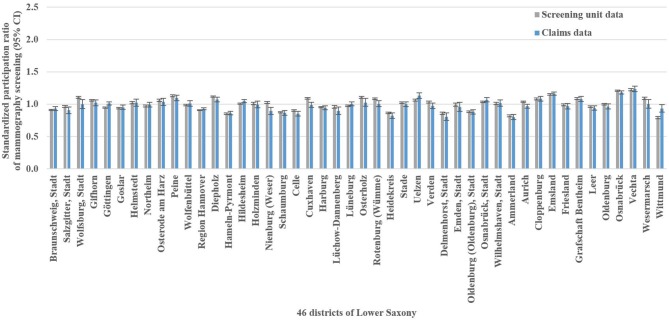
Screening unit and claims data-based standardized participation ratios of mammography screening with 95% confidence intervals (CI) in Lower Saxony between 2011 and 2014 for women aged 50–69 years.

The mammography screening analysis included 1,181,212 (screening unit data) and 147,325 (claims data) mammography screening events, respectively. In Lower Saxony, the overall population-based participation rate assessed in screening unit data was 57.5% (claims data: 58.9%). The amount of district-level variations assessed among the 46 districts of Lower Saxony using the benchmark data (i.e., screening unit data) is presented in Figure 1A. Screening unit data-based SPR ranged from 0.79 to 1.22 with a standard deviation (SD) of 0.10 and an interquartile range (IQR) of 0.13 (claims data: 0.80–1.24, SD 0.09, IQR 0.09). Statistically significant district-level variations were found for 39 (screening unit data) and 27 (claims data) districts, respectively (Figure 2, Supplementary Table 1).

In the Bland-Altman analysis, the claims-based SPR were between 0.13 below and 0.14 above the screening unit data-based SPR (**Figure 4A**). The differences between claims and screening unit data (y-axis) were unrelated to the averages of claims and screening unit data (x-axis). This implied that conventional 95% limits of agreement [LoA] could be calculated. The mean difference between claims and screening unit data was −0.02 and the conventional 95% LoA were −0.12 and 0.08.

### Standardized Incidence Ratios

**Figure 3:**
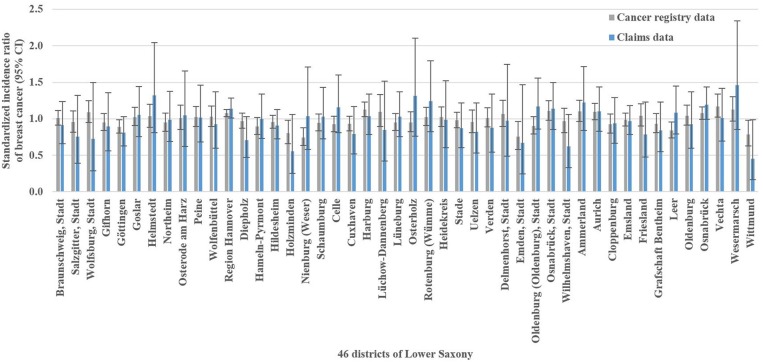
Cancer registry and claims data-based standardized incidence ratios of breast cancer with 95% confidence intervals (CI) in Lower Saxony between 2011 and 2014 for women aged 50–69 years.

The analysis of breast cancer incidence included 13,241 (cancer registry data) and 1,778 (claims data) incident breast cancer cases, respectively. The cancer registry data-based crude incidence in Lower Saxony was 322.3 per 100,000 women aged 50–69 years (claims data: 367.4 per 100,000 insured women in this age group). The amount of district-level variations assessed in the benchmark data (i.e., cancer registry data) is presented in Figure 1B. At the district level, cancer registry data-based SIR ranged from 0.75 to 1.17 with a SD of 0.10 and an IQR of 0.11 (claims data: 0.45–1.46, SD 0.20, IQR 0.24). Statistically significant district-level variations were found for 9 (cancer registry data) and 2 (claims data) districts, respectively (Figure 3, Supplementary Table 1).

**Figure 4:**
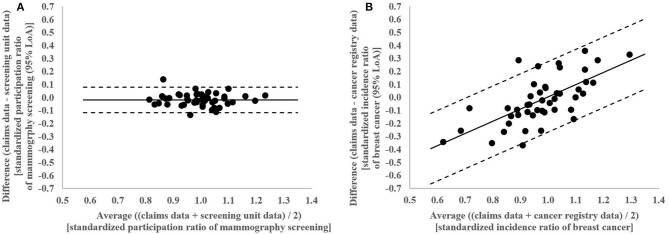
**(A)** Bland-Altman Plot of differences between claims and screening unit data plotted against the average of claims and screening unit data in standardized participation ratios of mammography screening in the 46 districts of Lower Saxony between 2011 and 2014 for women aged 50–69 years with conventional 95% limits of agreement (LoA). **(B)** Bland-Altman Plot of differences between claims and cancer registry data plotted against the average of claims and cancer registry data in standardized incidence ratios of breast cancer in the 46 districts of Lower Saxony between 2011 and 2014 for women aged 50–69 years with regression based 95% limits of agreement (LoA).

In the Bland-Altman analysis, the claims-based SIR were between 0.36 below and 0.36 above the cancer registry data-based SIR (Figure 4B). The differences between claims and cancer registry data tend to be negative for lower averages of claims and cancer registry data and positive for higher averages. This implied that regression based 95% LoA could be calculated. For the average of 1.0, the regression based mean difference was 0.00 and the regression based 95% LoA were −0.27 and 0.28.

### Univariable and Multilevel Logistic Regressions of Mammography Screening Participation

The univariable logistic regression analyses identified age, 24 out of the 31 Elixhauser comorbidities and all contextual-level variables to be associated with mammography screening participation (Supplementary Table 2). The variance inflation factor varied from 1.01 to 8.81 indicating no multicollinearity. In the multilevel analysis (*n* = 96,273), the MOR of 1.23 in the empty model indicated district-level differences (Table 1). Including individual-level variables in model 2 increased the MOR to 1.24. Compared to model 1, the proportional change in district-level variance was +5.3%. Additionally including contextual-level variables in model 3 decreased the MOR to 1.15. Compared to model 2, the proportional change in variance was −58.5%. Ten Elixhauer comorbidities and unemployment rate were negatively associated with mammography screening participation, and 11 Elixhauser comorbidities and the proportion of employees with an academic degree were positively associated.

**Table 1:** Multilevel logistic regression on the probability of participating in the German Mammography Screening Program between 2011 and 2014 for women aged 50–66 years in Lower Saxony (*n* = 96,273).

	**Model 1: empty model**		Model 2: + individual predictors^*^		Model 3: + contextual predictors^*^	
			**OR**	**95% CI**	**OR**	**95% CI**
**Age group (ref. 50–54 years)**						
55–59 years			1.00	(0.96–1.03)	1.00	(0.96–1.03)
60–64 years			1.00	(0.97–1.04)	1.00	(0.97–1.04)
65–66 years			0.98	(0.92–1.03)	0.98	(0.92–1.03)
**Elixhauser comorbidity (ref. no)**						
Congestive heart failure			**0.73**	**(0.68–0.80)**	**0.74**	**(0.68–0.80)**
Cardiac arrhythmias			**1.08**	**(1.03–1.14)**	**1.08**	**(1.03–1.14)**
Valvular disease			**1.22**	**(1.14–1.31)**	**1.22**	**(1.14–1.31)**
Pulmonary circulation disorders			0.90	(0.76–1.07)	0.90	(0.76–1.07)
Peripheral vascular disorders			**1.08**	**(1.01–1.15)**	**1.08**	**(1.01–1.15)**
Hypertension, uncomplicated			**1.24**	**(1.20–1.28)**	**1.24**	**(1.20–1.28)**
Hypertension, complicated			**1.47**	**(1.35–1.59)**	**1.47**	**(1.35–1.59)**
Paralysis			**0.54**	**(0.48–0.60)**	**0.54**	**(0.48–0.61)**
Other neurological disorders			**0.89**	**(0.82–0.96)**	**0.89**	**(0.82–0.96)**
Chronic pulmonary disease			**1.13**	**(1.10–1.17)**	**1.13**	**(1.10–1.17)**
Diabetes, uncomplicated			**0.85**	**(0.80–0.90)**	**0.85**	**(0.80–0.90)**
Hypothyroidism			**1.34**	**(1.29–1.40)**	**1.34**	**(1.29–1.40)**
Renal failure			**0.84**	**(0.76–0.92)**	**0.84**	**(0.76–0.92)**
Liver disease			**1.07**	**(1.01–1.12)**	**1.07**	**(1.01–1.12)**
Metastatic cancer			**0.60**	**(0.53–0.68)**	**0.60**	**(0.53–0.68)**
Solid tumor without metastasis			**1.12**	**(1.03–1.21)**	**1.11**	**(1.03–1.21)**
Rheumatoid arthritis/collagen vascular diseases			**1.38**	**(1.31–1.46)**	**1.38**	**(1.31–1.46)**
Coagulopathy			0.97	(0.87–1.08)	0.97	(0.87–1.08)
Obesity			**1.21**	**(1.16–1.26)**	**1.21**	**(1.16–1.26)**
Weight loss			**0.76**	**(0.68–0.86)**	**0.76**	**(0.68–0.86)**
Fluid and electrolyte disorders			**0.73**	**(0.67–0.79)**	**0.73**	**(0.67–0.79)**
Alcohol abuse			0.94	(0.89–1.00)	0.95	(0.89–1.00)
Drug abuse			**0.52**	**(0.45–0.60)**	**0.52**	**(0.45–0.60)**
Psychoses			**0.56**	**(0.51–0.62)**	**0.56**	**(0.51–0.62)**
**Unemployment rate (ref. quintile 1)**						
Quintile 2					**0.75**	**(0.61–0.92)**
Quintile 3					**0.77**	**(0.63–0.94)**
Quintile 4					**0.72**	**(0.59–0.88)**
Quintile 5					**0.67**	**(0.54–0.84)**
**Average household income per inhabitant (ref. quintile 1)**						
Quintile 2					0.91	(0.74–1.11)
Quintile 3					1.04	(0.86–1.26)
Quintile 4					0.88	(0.70–1.10)
Quintile 5					0.95	(0.76–1.18)
**Proportion of employees without a qualification (ref. quintile 1)**						
Quintile 2					0.89	(0.74–1.08)
Quintile 3					1.02	(0.82–1.25)
Quintile 4					1.00	(0.84–1.20)
Quintile 5					1.14	(0.93–1.39)
**Proportion of employees with an academic degree (ref. quintile 1)**						
Quintile 2					**1.22**	**(1.01–1.47)**
Quintile 3					1.17	(0.98–1.40)
Quintile 4					1.17	(0.98–1.41)
Quintile 5					**1.36**	**(1.08–1.70)**
**Proportion of foreign population (ref. quintile 1)**						
Quintile 2					0.84	(0.68–1.04)
Quintile 3					0.96	(0.80–1.15)
Quintile 4					1.08	(0.90–1.28)
Quintile 5					1.06	(0.89–1.27)
**Type of district (ref. large cities)**						
urban cities					0.98	(0.78–1.23)
urban-rural districts					1.07	(0.82–1.39)
rural districts					1.25	(0.94–1.66)
**Measures of variation**						
district-level variance (SE)	0.047 (0.010)		0.049 (0.011)		0.020 (0.005)	
proportional change in variance			+5.3%		−58.5%	
MOR	1.23		1.24		1.15	
**Fit statistics**						
−2 log likelihood	119516.5		116206.9^**^		116171.9^**^	

*ref, reference; SE, standard error; MOR, median odds ratio; OR, odds ratio; CI, confidence interval*.

Boldface indicates statistical significance.

* *Controlled for different observation times*.

** *Significant likelihood ratio test*.

## Discussion

For the first time, we systematically examined the suitability of German health insurance claims data for assessing and explaining district-level variations in mammography screening participation and breast cancer incidence using screening unit, cancer registry and health insurance claims data. Among the 46 districts of Lower Saxony, we found significant district-level variations in mammography screening participation and hardly any district-level variations in breast cancer incidence. We found geographic variations in mammography screening participation assessed in health insurance claims data to be comparable with those assessed in screening unit data. Therefore, we hold that health insurance claims data are suitable for assessing district-level variations in mammography screening participation. The agreement between health insurance claims and cancer registry data was considerably lower, so that we consider the suitability of health insurance claims data for assessing district-level variations in breast cancer incidence to be limited. The difference in suitability can be explained by the fact that the precision of our district-level estimates was high for mammography screening participation with high numbers of mammography screening events and low for breast cancer incidence with low numbers of incident breast cancer cases.

Because health insurance claims data appear to be suitable for assessing geographic variations in mammography screening participation at the district level, they might also be useful for explaining respective variations. In our analysis, we investigated the effect of the individual-level variables age and comorbidity as well as the contextual-level variables unemployment, income, education, foreign population and type of district on mammography screening participation and the role of these variables in explaining district-level variations in mammography screening participation. At the individual level, age was not associated with mammography screening participation. Previous studies including women younger than 50 and older than 69 years, however, found associations for age (34, 35, 49–51). For comorbidity, we observed diverse associations with mammography screening participation. While strong negative associations were found for drug abuse, paralysis, psychoses and metastatic cancer, strong positive associations were found for hypertension, rheumatoid arthritis/collagen vascular diseases and hypothyroidism. These findings seem plausible since severe morbidities, such as mental and neurological diseases as well as metastatic cancer may inhibit mammography screening participation (52–55). Diagnoses with less severe morbidities, by contrast, may result in an increased health awareness and thus an increased likelihood of participating in mammography screening.

At the contextual level, in line with previous studies (24, 35), we found unemployment to be negatively associated with mammography screening participation. A positive association was found for the proportion of employees with an academic degree which is in line with previous studies that found similar associations for education (56, 57).

We found that the individual-level variables age and comorbidity did not contribute to the explanation of district-level variations in mammography screening participation. This was indeed the case for the contextual-level variables unemployment, income, education, foreign population and type of district which explained more than half of the assessed spatial disparities. The remaining geographic variations must be due to other determinants of mammography screening such as lifestyle factors (58), cancer anxiety and worry (56, 59, 60), emotional barriers against mammography screening (60, 61) (informed), decisions for or against mammography screening (62) and accessibility of mammography screening (63), for which we had no information. Since mammography screening participation rates are about 5 percentage points higher when screening mammograms coded as diagnostic mammograms are also considered (64), geographic variations in diagnostic mammography could also play a role.

### Strengths and Limitations

This is the first study demonstrating the suitability of health insurance claims data for assessing geographic variations in mammography screening participation at the district level. Furthermore, we demonstrated for the first time that individual health insurance claims data linked to contextual data for both mammography screening participants and non-participants offer the potential to explain a considerable part of the existing district-level variations in mammography screening participation.

There are, however, some important limitations to consider. First, to increase the precision of our district-level estimates, we assessed district-level variations in mammography screening participation and breast cancer incidence over a 4-years period and did not consider small changes over time. Second, to identify incident breast cancer cases, we applied a claims-based algorithm and not a linkage of health insurance claims data and cancer registry data. Different definitions of the algorithm might have resulted in different district-level estimates for breast cancer incidence. Third, in the multilevel analysis of mammography screening participation, we were not able to separate individual from contextual socioeconomic effects since we lacked individual-level data on socioeconomic characteristics. Fourth, we assessed individual- and contextual-level determinants in a given period and did not consider changes over time. Fifth, our results are limited by the Modifiable Area Unit Problem (65). Analyzing other geographic units might have led to different results. Finally, we used claims data from only one of the currently 109 German statutory health insurance funds insuring 90% of the German population and did not consider claims data from the private health insurance insuring 10% of the German population. Both aspects limit the precision of our claims-based district-level estimates and the generalizability of our findings (37, 38).

### Conclusions

Mammography screening participation differs at the district level whereas hardly any district-level variations exist for breast cancer incidence. Health insurance claims data are suitable for assessing and explaining geographic variations in mammography screening participation but their suitability for assessing and explaining geographic variations in breast cancer incidence appears to be limited. To fully exploit the potential of health insurance claims data for investigating individual- and contextual-level determinants of mammography screening, future studies should consider claims data of several health insurance funds and combine these data with information on individual-level socioeconomic characteristics, lifestyle factors, psychological factors, quality of life and health literacy as well as on contextual-level socioeconomic characteristics and accessibility to mammography screening. This allows a comprehensive investigation of geographic variations in mammography screening participation and helps to identify indications to further improve prevention strategies for reducing the burden of breast cancer.

## Data Availability

The health insurance claims, screening unit, and cancer registry data were obtained from the BARMER, the eight screening units located in the federal state of Lower Saxony, and the Epidemiological Cancer Registry of Lower Saxony. These data were analyzed under license for the study. Requests to access these data should be directed to the BARMER, the screening units located in Lower Saxony, and the Epidemiological Cancer Registry of Lower Saxony. The contextual data were obtained from the Federal Institute for Research on Building, Urban Affairs, and Spatial Development. These data are publicly available.

## Ethics Statement

This study considered pseudonymous health insurance claims data, anonymous screening unit data, and anonymous cancer registry data. The use of these data was approved by the responsible authorities (i.e., BARMER, Reference Center for Mammography Nord, and Epidemiological Cancer Registry of Lower Saxony, respectively). An ethics statement was not required by law.

## Author Contributions

JC and FH conceptualized the study. IU, JK, FS, and IL contributed to the design of the study. JC and FH acquired the health insurance claims data. IU and JK acquired the screening unit and cancer registry data. JC and FS acquired the contextual data. JC and FH analyzed the health insurance claims data. JC and IU analyzed the screening unit and cancer registry data. JC wrote the first draft of the manuscript. The manuscript was critically revised by IU, JK, FS, IL, and FH. All authors approved the final version of the manuscript.

### Conflict of Interest Statement

The authors declare that the research was conducted in the absence of any commercial or financial relationships that could be construed as a potential conflict of interest. The reviewer AB declared a past collaboration with one of the authors JK to the handling editor.
