# Supplementary material for: Assessing and Explaining Geographic Variations in Mammography Screening Participation and Breast Cancer Incidence
Source: Front Oncol. 2019 Sep 18;9:909. doi: 10.3389/fonc.2019.00909 (PMC6759661; doi:10.3389/fonc.2019.00909)
Supplement: Supplementary file 1 [file Data_Sheet_1.PDF]

Supplementary Material

Identification of the individual Elixhauser comorbidities

The individual Elixhauser comorbidities were identified on the basis of outpatient diagnoses as well as on main and secondary hospital discharge diagnoses in 2011 and 2012 (i.e. in the first of two biennial invitation rounds). ICD-10 codes provided by Quan et al. 2015 (1) were considered with the following modifications applied:

- Exclusion of breast cancer diagnoses (ICD-10 C50) from the category “solid tumor without metastasis”.
- Consideration of only “hypertension, complicated” for women who had both “hypertension, uncomplicated” and “hypertension, complicated”.
- Consideration of only “metastatic cancer” for women who had both “solid tumor without metastasis” and “metastatic cancer”.
- Consideration of only “diabetes, complicated” for women who had “diabetes, uncomplicated” and “diabetes complicated”.

Description of contextual data

The **unemployment rate** was publicly available from the Federal Institute for Research on Building, Urban Affairs and Spatial Development (BBSR) [2] and defined as the proportion of unemployed people among the total labor force. It was assessed for the 46 districts of Lower Saxony in 2011 and categorized into the following quintiles:

- Quintile 1: ≤5.0%
- Quintile 2: >5.0% to ≤6.5%
- Quintile 3: >6.5% to ≤7.6%
- Quintile 4: >7.6% to ≤8.3%
- Quintile 5: >8.3%

The **average household income per inhabitant** was publicly available from the BBSR [2] and defined as the monthly mean net household income in € per inhabitant. It was assessed for the 46 districts of Lower Saxony in 2011 and categorized into the following quintiles:

- Quintile 1: ≤1,507€
- Quintile 2: >1,507€ to ≤1,540€
- Quintile 3: >1,540€ to ≤1,594€
- Quintile 4: >1,594€ to ≤1,647€
- Quintile 5: >1,647€

The **proportion of employees without a qualification** was publicly available from the BBSR [2] and defined as the proportion of employees without a degree covered by social insurance among the total population of employees covered by social insurance. Because data for the year 2011 were not available, it was assessed for the 46 districts of Lower Saxony in 2012. The variable was categorized into the following quintiles:

- Quintile 1: ≤11.3%
- Quintile 2: >11.3% to ≤12.4%
- Quintile 3: >12.4% to ≤12.8%
- Quintile 4: >12.8% to ≤13.5%
- Quintile 5: >13.5%

The **proportion of employees with an academic degree** was publicly available from the BBSR [2] and defined as the proportion of employees with an academic degree covered by social insurance among the total population of employees covered by social insurance. Because data for the year 2011 were not available, it was assessed for the 46 districts of Lower Saxony in 2012. The variable was categorized into the following quintiles:

- Quintile 1: ≤6.0%
- Quintile 2: >6.0% to ≤6.8%
- Quintile 3: >6.8% to ≤7.3%
- Quintile 4: >7.3% to ≤9.5%
- Quintile 5: >9.5%

The **proportion of foreign population** was publicly available from the BBSR [2] and defined as the proportion of foreigners among the total population of inhabitants. It was assessed for the 46 districts of Lower Saxony in 2011 and categorized into the following quintiles:

- Quintile 1: ≤3.4%
- Quintile 2: >3.4% to ≤4.2%
- Quintile 3: >4.2% to ≤4.7%
- Quintile 4: >4.7% to ≤6.0%
- Quintile 5: >6.0%

The **type of district** was publicly available from the BBSR [2]. Because data for the year 2011 were not available, it was assessed for the 46 districts of Lower Saxony in 2015. The variable comprised the following categories:

- Large cities
- Urban cities
- Urban-rural districts
- Rural districts

Supplementary Table 1. Screening unit and claims data-based standardized participation ratios of mammography screening with 95% confidence intervals as well as cancer registry and claims data-based standardized incidence ratios of breast cancer with 95% confidence intervals in Lower Saxony between 2011 and 2014 for women aged 50 to 69 years.

	SPR (95% CI)		SIR (95% CI)	
**District**	**Screening unit data**	**Claims data**	**Cancer registry data**	**Claims data**
Braunschweig, Stadt	**0.91 (0.90-0.92)**	**0.93 (0.90-0.96)**	1.01 (0.91-1.11)	0.91 (0.66-1.24)
Salzgitter, Stadt	**0.97 (0.95-0.98)**	**0.91 (0.86-0.96)**	0.96 (0.82-1.11)	0.76 (0.39-1.32)
Wolfsburg, Stadt	**1.11 (1.09-1.12)**	1.00 (0.93-1.07)	1.09 (0.95-1.25)	0.73 (0.29-1.50)
Gifhorn	**1.06 (1.05-1.07)**	1.02 (0.98-1.07)	0.95 (0.84-1.07)	0.90 (0.56-1.36)
Göttingen	**0.95 (0.94-0.96)**	1.01 (0.99-1.04)	**0.89 (0.80-0.99)**	0.81 (0.63-1.03)
Goslar	**0.94 (0.93-0.95)**	**0.95 (0.92-0.99)**	1.03 (0.91-1.16)	1.06 (0.76-1.44)
Helmstedt	**1.02 (1.01-1.04)**	1.02 (0.97-1.08)	1.03 (0.88-1.20)	1.32 (0.81-2.04)
Northeim	**0.97 (0.96-0.98)**	0.99 (0.96-1.03)	0.95 (0.83-1.08)	0.99 (0.69-1.37)
Osterode am Harz	**1.06 (1.04-1.08)**	1.04 (0.98-1.09)	1.01 (0.85-1.19)	1.05 (0.62-1.66)
Peine	**1.13 (1.12-1.15)**	**1.09 (1.05-1.14)**	1.03 (0.90-1.17)	1.02 (0.68-1.46)
Wolfenbüttel	0.99 (0.97-1.00)	1.01 (0.97-1.05)	1.03 (0.90-1.17)	0.93 (0.60-1.37)
Region Hannover	**0.91 (0.90-0.91)**	**0.93 (0.92-0.94)**	**1.08 (1.03-1.12)**	**1.14 (1.01-1.28)**
Diepholz	**1.12 (1.11-1.13)**	**1.07 (1.04-1.11)**	0.97 (0.87-1.08)	0.71 (0.47-1.03)
Hameln-Pyrmont	**0.85 (0.84-0.86)**	**0.86 (0.83-0.89)**	0.90 (0.79-1.02)	1.00 (0.73-1.34)
Hildesheim	1.01 (1.00-1.02)	**1.05 (1.02-1.07)**	0.96 (0.87-1.05)	0.91 (0.72-1.12)
Holzminden	1.01 (0.99-1.03)	0.99 (0.94-1.05)	**0.81 (0.66-0.98)**	0.56 (0.25-1.06)
Nienburg (Weser)	**1.03 (1.01-1.04)**	**0.89 (0.84-0.95)**	**0.75 (0.63-0.88)**	1.04 (0.58-1.71)
Schaumburg	**0.88 (0.86-0.89)**	**0.87 (0.83-0.90)**	0.94 (0.83-1.07)	1.03 (0.72-1.43)
Celle	**0.90 (0.89-0.91)**	**0.85 (0.82-0.89)**	0.92 (0.82-1.04)	1.16 (0.81-1.60)
Cuxhaven	**1.09 (1.08-1.10)**	0.99 (0.95-1.03)	0.93 (0.84-1.04)	0.80 (0.52-1.17)
Harburg	**0.96 (0.95-0.97)**	**0.94 (0.92-0.97)**	**1.13 (1.03-1.23)**	1.03 (0.78-1.34)
Lüchow-Dannenberg	**0.96 (0.94-0.98)**	**0.90 (0.84-0.96)**	1.10 (0.90-1.33)	0.85 (0.42-1.52)
Lüneburg	**0.97 (0.96-0.99)**	1.00 (0.97-1.04)	0.95 (0.84-1.07)	1.03 (0.76-1.37)
Osterholz	**1.11 (1.09-1.12)**	1.03 (0.97-1.09)	0.95 (0.82-1.10)	1.31 (0.76-2.10)
Rotenburg (Wümme)	**1.09 (1.07-1.10)**	1.01 (0.96-1.05)	1.02 (0.91-1.15)	1.24 (0.82-1.79)
Heidekreis	**0.87 (0.85-0.88)**	**0.82 (0.78-0.87)**	1.02 (0.90-1.16)	0.98 (0.60-1.52)
Stade	**1.02 (1.01-1.04)**	1.00 (0.97-1.03)	0.98 (0.88-1.09)	0.87 (0.60-1.22)
Uelzen	**1.06 (1.05-1.08)**	**1.13 (1.09-1.17)**	0.96 (0.81-1.12)	0.82 (0.53-1.22)
Verden	**1.03 (1.02-1.05)**	0.97 (0.93-1.02)	1.01 (0.89-1.15)	0.88 (0.54-1.34)
Delmenhorst, Stadt	**0.86 (0.84-0.87)**	**0.80 (0.75-0.86)**	1.07 (0.90-1.26)	0.98 (0.49-1.75)
Emden, Stadt	0.99 (0.97-1.02)	0.96 (0.89-1.03)	**0.75 (0.58-0.96)**	0.67 (0.25-1.47)
Oldenburg (Oldenburg), Stadt	**0.88 (0.87-0.90)**	**0.88 (0.85-0.91)**	0.90 (0.79-1.03)	1.17 (0.86-1.56)
Osnabrück, Stadt	**1.04 (1.02-1.05)**	**1.07 (1.03-1.10)**	1.11 (0.98-1.25)	1.14 (0.85-1.50)
Wilhelmshaven, Stadt	1.01 (0.99-1.03)	1.02 (0.97-1.07)	0.97 (0.81-1.15)	0.62 (0.33-1.06)
Ammerland	**0.82 (0.81-0.83)**	**0.80 (0.76-0.84)**	1.10 (0.96-1.25)	1.22 (0.84-1.72)
Aurich	**1.04 (1.03-1.05)**	**0.97 (0.94-1.00)**	1.09 (0.99-1.21)	1.10 (0.83-1.43)
Cloppenburg	**1.08 (1.07-1.09)**	**1.09 (1.05-1.12)**	0.93 (0.81-1.06)	0.94 (0.67-1.29)
Emsland	**1.15 (1.14-1.16)**	**1.16 (1.14-1.19)**	0.99 (0.90-1.08)	0.97 (0.79-1.18)
Friesland	0.99 (0.98-1.01)	0.97 (0.93-1.01)	1.04 (0.90-1.20)	0.79 (0.47-1.23)
Grafschaft Bentheim	**1.09 (1.07-1.10)**	**1.08 (1.04-1.12)**	0.93 (0.81-1.07)	0.84 (0.55-1.23)
Leer	**0.96 (0.95-0.98)**	**0.94 (0.91-0.97)**	**0.84 (0.74-0.96)**	1.09 (0.79-1.45)
Oldenburg	1.00 (0.99-1.01)	0.97 (0.93-1.01)	1.04 (0.91-1.19)	0.93 (0.60-1.37)
Osnabrück	**1.21 (1.20-1.22)**	**1.18 (1.16-1.20)**	1.08 (0.99-1.17)	1.20 (0.99-1.43)
Vechta	**1.22 (1.21-1.24)**	**1.24 (1.20-1.28)**	**1.17 (1.02-1.34)**	1.01 (0.69-1.42)
Wesermarsch	**1.09 (1.07-1.11)**	1.00 (0.94-1.07)	1.13 (0.97-1.31)	1.46 (0.85-2.34)
Wittmund	**0.79 (0.77-0.81)**	**0.93 (0.88-0.99)**	**0.79 (0.63-0.98)**	**0.45 (0.17-0.98)**

Abbreviations: SPR, standardized participation ratio; SIR, standardized incidence ratio; CI, confidence interval.
Boldface indicates statistical significance.

Supplementary Table 2. Univariable logistic regressions on the probability of participating in the German Mammography Screening Program between 2011 and 2014 for women aged 50 to 66 years in Lower Saxony.

	OR	95% CI	p-value
Age group (ref. 50-54 years)			
55-59 years	**1.04**	**(1.00-1.07)**	**0.0418**
60-64 years	**1.06**	**(1.02-1.10)**	**0.0012**
65-66 years	**1.03**	**(0.98-1.09)**	**0.2430**
Elixhauser comorbidity (ref. no)			
Congestive heart failure	**0.82**	**(0.76-0.88)**	**<.0001**
Cardiac arrhythmias	**1.17**	**(1.12-1.22)**	**<.0001**
Valvular disease	**1.23**	**(1.16-1.32)**	**<.0001**
Pulmonary circulation disorders	**0.78**	**(0.67-0.91)**	**0.0017**
Peripheral vascular disorders	**1.06**	**(1.00-1.12)**	**0.0635**
Hypertension, uncomplicated	**1.24**	**(1.21-1.28)**	**<.0001**
Hypertension, complicated	**1.30**	**(1.21-1.39)**	**<.0001**
Paralysis	**0.47**	**(0.42-0.52)**	**<.0001**
Other neurological disorders	**0.69**	**(0.64-0.74)**	**<.0001**
Chronic pulmonary disease	**1.18**	**(1.14-1.22)**	**<.0001**
Diabetes, uncomplicated	**0.94**	**(0.89-1.00)**	**0.0319**
Diabetes, complicated	1.01	(0.94-1.09)	0.7896
Hypothyroidism	**1.38**	**(1.32-1.43)**	**<.0001**
Renal failure	**0.82**	**(0.75-0.90)**	**<.0001**
Liver disease	**1.09**	**(1.04-1.15)**	**0.0005**
Peptic ulcer disease excluding bleeding	1.04	(0.90-1.20)	0.6097
AIDS/HIV	0.84	(0.37-1.81)	0.6493
Lymphoma	0.89	(0.74-1.08)	0.2474
Metastatic cancer	**0.39**	**(0.35-0.43)**	**<.0001**
Solid tumor without metastasis	**1.11**	**(1.03-1.20)**	**0.008**
Rheumatoid arthritis/collagen vascular diseases	**1.45**	**(1.37-1.52)**	**<.0001**
Coagulopathy	**0.79**	**(0.72-0.87)**	**<.0001**
Obesity	**1.30**	**(1.26-1.35)**	**<.0001**
Weight loss	**0.59**	**(0.53-0.65)**	**<.0001**
Fluid and electrolyte disorders	**0.60**	**(0.56-0.64)**	**<.0001**
Blood loss anemia	0.89	(0.72-1.08)	0.2343
Deficiency anemia	0.99	(0.92-1.06)	0.7904
Alcohol abuse	**0.89**	**(0.84-0.93)**	**<.0001**
Drug abuse	**0.48**	**(0.41-0.55)**	**<.0001**
Psychoses	**0.53**	**(0.48-0.59)**	**<.0001**
Depression	1.01	(0.98-1.04)	0.4995
Unemployment rate (ref. quintile 1)			
Quintile 2	**0.84**	**(0.80-0.88)**	**<.0001**
Quintile 3	**0.83**	**(0.79-0.86)**	**<.0001**
Quintile 4	**0.76**	**(0.73-0.79)**	**<.0001**
Quintile 5	**0.66**	**(0.64-0.69)**	**<.0001**
Average household income per inhabitant (ref. quintile 1)			
Quintile 2	0.98	(0.94-1.03)	0.4639
Quintile 3	0.99	(0.94-1.04)	0.6204
Quintile 4	**0.91**	**(0.87-0.95)**	**<.0001**
Quintile 5	1.03	(0.98-1.08)	0.3355
Proportion of employees without a qualification (ref. quintile 1)			
Quintile 2	1.01	(0.97-1.05)	0.6896
Quintile 3	**1.06**	**(1.00-1.11)**	**0.0329**
Quintile 4	**1.11**	**(1.05-1.16)**	**<.0001**
Quintile 5	**1.40**	**(1.32-1.47)**	**<.0001**

Supplementary Table 2. (continued).

	OR	95% CI	p-value
Proportion of employees with an academic degree (ref. quintile 1)			
Quintile 2	**1.18**	**(1.13-1.24)**	**<.0001**
Quintile 3	**1.18**	**(1.12-1.24)**	**<.0001**
Quintile 4	**0.93**	**(0.89-0.98)**	**0.0069**
Quintile 5	**0.93**	**(0.89-0.97)**	**0.0011**
Proportion of foreign population (ref. quintile 1)			
Quintile 2	0.98	(0.93-1.03)	0.4698
Quintile 3	**1.15**	**(1.10-1.21)**	**<.0001**
Quintile 4	**1.10**	**(1.05-1.15)**	**<.0001**
Quintile 5	**0.93**	**(0.89-0.97)**	**0.0014**
Type of district (ref. large cities)			
urban cities	1.02	(0.97-1.08)	0.3808
urban-rural districts	**1.23**	**(1.17-1.30)**	**<.0001**
rural districts	**1.23**	**(1.17-1.30)**	**<.0001**

Abbreviations: ref., reference; OR, odds ratio; CI, confidence interval.
Boldface indicates p-values < 0.2.

References (Supplementary Material)

1. Quan H, Sundararajan V, Halfon P, Fong A, Burnand B, Luthi JC, et al. Coding algorithms for defining comorbidities in ICD-9-CM and ICD-10 administrative data. *Med Care* (2005) 43:1130-9. doi: 10.1097/01.mlr.0000182534.19832.83

2. Federal Institute for Research on Building, Urban Affairs and Spatial Development, editor. Indicators and maps on spatial and urban development. INKAR. Volume 2018. Bonn: Federal Institute for Research on Building, Urban Affairs and Spatial Development within the Federal Office for Building and Regional Planning. Available online at: https://www.inkar.de (Accessed January 19, 2019).
